# Holmium laser ureterocele excision with transurethral incision of the prostate

**DOI:** 10.1590/S1677-5538.IBJU.2020.0464

**Published:** 2021-03-10

**Authors:** Grant R. Pollock, Kalpesh Patel, Joel Funk

**Affiliations:** 1 University of Arizona College of Medicine Tucson Department of Urology TucsonAZ USA Department of Urology, University of Arizona College of Medicine Tucson, Tucson, AZ, USA.; 2 Arizona Institute of Urology TucsonAZ USA Arizona Institute of Urology, Tucson, AZ, USA.

## Abstract

**Introduction:**

Ureteroceles present a diagnostic and treatment challenge in adults (1). With an estimated prevalence of 1/500 to 1/4000, it is not uncommon for any urologist to encounter ureteroceles in clinical practice (2). The incidence of prolapsed ureteroceles in adults is unknown (3).

**Materials and Methods:**

We present an interesting case of a 53-year-old male with a 20-year history of obstructive voiding symptoms who presented with urinary retention with a Foley catheter in place. Pre-operative evaluation included a transrectal ultrasound of the prostate which revealing prostate volume of 20cc. Urodynamics revealed a high-pressure, low flow voiding pattern with a functional detrusor muscle. Cystourethroscopy was performed revealing an orthotopic ureterocele on the left side that was prolapsed into the prostatic urethra, and the bladder neck was elevated. The patient then underwent holmium laser ureterocele excision with transurethral incision of the prostate (TUIP). Using MOSES technology and laser settings of 30Hz and 1.5J, the ureterocele was completely excised and a TUIP was performed.

**Results:**

The patient was discharged home on the day of surgery with a Foley catheter in place. On post-operative day 1 he passed a voiding trial with a post-void residual volume of 25cc. Renal ultrasonography was performed 3 months postoperatively revealing no hydronephrosis. His postoperative International Prostate Symptom Score of 2 was improved compared to his preoperative score of 34.

**Conclusion:**

Holmium laser ureterocele excision with a TUIP is an effective treatment modality in the management of a prolapsed orthotopic ureterocele causing bladder outlet obstruction in a male patient.

## ABBREVIATIONS

TUIP: Transurethral Incision of the Prostate
